# Tumoral PD-L1 expression defines a subgroup of poor-prognosis vulvar carcinomas with non-viral etiology

**DOI:** 10.18632/oncotarget.21641

**Published:** 2017-10-06

**Authors:** Thomas Hecking, Thore Thiesler, Cynthia Schiller, Jean-Marc Lunkenheimer, Tiyasha H. Ayub, Andrea Rohr, Mateja Condic, Mignon-Denise Keyver-Paik, Rolf Fimmers, Jutta Kirfel, Walther Kuhn, Glen Kristiansen, Kirsten Kübler

**Affiliations:** ^1^ Department of Obstetrics and Gynecology, Center for Integrated Oncology, University of Bonn, Bonn, Germany; ^2^ Institute of Pathology, Center for Integrated Oncology, University of Bonn, Bonn, Germany; ^3^ Institute of Medical Biometry, Informatics and Epidemiology, Center for Integrated Oncology, University of Bonn, Bonn, Germany; ^4^ Hospital of Augustinian Nuns, Cologne, Germany; ^5^ Ärzte am Bärenplatz, Hornberg, Germany; ^6^ Broad Institute of MIT and Harvard, Cambridge, MA, USA; ^7^ Center for Cancer Research, Massachusetts General Hospital, Boston, MA, USA; ^8^ Harvard Medical School, Boston, MA, USA

**Keywords:** vulvar cancer, PD-L1, immune checkpoint, prognostic factor, HPV

## Abstract

Vulvar cancer is rare but incidence rates are increasing due to an aging population and higher frequencies of young women being affected. In locally advanced, metastatic or recurrent disease prognosis is poor and new treatment modalities are needed. Immune checkpoint blockade of the PD-1/PD-L1 pathway is one of the most important advancements in cancer therapy in the last years. The clinical relevance of PD-L1 expression in vulvar cancer, however, has not been studied so far. We determined PD-L1 expression, numbers of CD3^+^ T cells, CD20^+^ B cells, CD68^+^ monocytes/macrophages, Foxp3^+^ regulatory T cells and CD163^+^ tumor-associated macrophages by immunohistochemistry in 103 patients. Correlation analysis with clinicopathological parameters was undertaken; the cause-specific outcome was modeled with competing risk analysis; multivariate Cox regression was used to determine independent predictors of survival. Membranous PD-L1 was expressed in a minority of tumors, defined by HPV-negativity. Its presence geographically correlated with immunocyte-rich regions of cancer islets and was an independent prognostic factor for poor outcome. Our data support the notion that vulvar cancer is an immunomodulatory tumor that harnesses the PD-1/PD-L1 pathway to induce tolerance. Accordingly, immunotherapeutic approaches might have the potential to improve outcome in patients with vulvar cancer and could complement conventional cancer treatment.

## INTRODUCTION

Vulvar cancer - with squamous cell carcinoma (VSCC) being the predominant histologic subtype - is only the fourth most common tumor of the female genital tract, accounting for 5% of all gynecologic malignancies in developed countries [1]. However, overall incidence rates are increasing, mainly attributable to a strong rise in younger women [2–4]. Etiology divides VSCC into two subtypes: (i) human papillomavirus (HPV)-associated tumors, typically diagnosed in younger women; and (ii) tumors arising in the background of *lichen sclerosus et atrophicus*, mostly seen in advanced age [5]. Approximately 65% of VSCCs are HPV-positive and the oncogenic high-risk types HPV-16/-18/-31/-33 are most frequently detected [6]. However, the burden of VSCC still lies with older women, in whom survival rates are low due to more aggressive tumors, diagnosis at later stages and frequent under-treatment [7, 8]. Surgery - consisting of local tumor excision with sentinel or inguinofemoral lymph node dissection - is considered the cornerstone of therapy. It leads to an excellent 5-year survival rate of 86% in localized VSCC [9]. Patients with locally advanced, metastatic or recurrent disease are additionally treated with radio- and chemotherapy. But the outcome is still poor and new therapeutic options are needed to reduce mortality in these women [10].

The recent success of checkpoint blockade caused a paradigm shift in cancer treatment. It focuses on disinhibiting tumor-specific immune responses. One of the most important immune-inhibitory checkpoint pathways consists of (i) the programmed death 1 (PD-1) receptor, expressed on activated T cells; and (ii) the programmed death ligand 1 (PD-L1), expressed on tumor cells [11]. Their interaction negatively regulates T cell proliferation and function. This process allows the tumor to evade immune detection. PD-L1 levels were found to be present on various solid cancer types, typically conferring a poor prognosis [12–15]. Blockade of the PD-1/PD-L1 signaling pathway was demonstrated to enhance T cell function and has been successfully used in the treatment of multiple tumor types [16]. To bring attention to the opportunity checkpoint blockade might offer to VSCC, we here evaluate the clinical impact of PD-L1 expression. Our approach is motivated by two observations. First, VSCC is able to provoke a tumor-specific immune response. Both pro-inflammatory subsets and immunosuppressive mechanisms have been described [17–19]. Second, the clinical response to immune checkpoint blockade is correlated with tumor PD-L1 expression across multiple malignancies [20]. To form the basis for future use of checkpoint inhibitors in VSCC we here aim to analyze (i) the expression status of PD-L1; (ii) its relation to tumor biology; and (iii) its prognostic value.

## RESULTS

### VSCC is characterized by an immune-active tumor microenvironment

We first evaluated whether VSCC is a malignancy able to elicit an inflammatory response using samples that cover the wide range of the disease (Table 1). Several cellular components of the immune system were evaluated in cancer cell nests and the stroma separately. We found CD3^+^ T cells, CD20^+^ B cells and CD68^+^ monocytes/macrophages to be present in both compartments (Table 2). Immunosuppressive cell populations analyzed included Foxp3^+^ regulatory T cells (T_reg_ cells) and CD163^+^ tumor-associated macrophages (TAMs), shown to play a crucial role in various cancer types [21, 22]. These anti-inflammatory cell types also existed in both the tumor and the stroma (Supplementary Table 1). For all immune cell types analyzed frequencies in the stroma predominated over numbers in tumor islets.

**Table 1:** Clinicopathological patient characteristics

Variable		Value [mean ± SD (range)]
Age (yrs)		64 ± 15 (26 - 93)
**Variable**		**Value [median (95% CI)]**
Follow-up time (ms)		46.7 (31.1 - 55.8)
**Variable**		**Value** [n (%)]
Tumor stage	pT1a	16 (16)
	pT1b	67 (65)
	pT2	15 (14)
	pT3	1 (1)
	ND	4 (4)
Depth of stromal invasion	≤ 1mm	12 (12)
	> 1mm	75 (73)
	ND	16 (15)
Lymph node involvement	Present	31 (30)
	Absent	40 (39)
	ND	32 (31)
Metastasis	Present	5 (5)
	Absent	94 (91)
	ND	4 (4)
Tumor grade	1	12 (12)
	2	66 (64)
	3	24 (23)
	ND	1 (1)
Lymphovascular space invasion	Present	8 (8)
	Absent	80 (78)
	ND	15 (14)
Haemangioinvasion	Present	6 (6)
	Absent	82 (80)
	ND	15 (14)
Local surgical treatment	Wide local excision	15 (15)
	Partial vulvectomy	35 (34)
	Vulvectomy	49 (47)^*^
	Punch biopsy only	4 (4)^**^
Tumor-free margins	Present	86 (84)
	Absent	17 (16)
Lymph node dissection	Sentinel	4 (4)
	Inguinal (uni-, bilateral)	66 (64)^***^
	No lymph node dissection	33 (32)
Radiation therapy	Vulvar	12 (12)
	Inguinal	8 (8)
	Vulvar & inguinal	22 (21)^****^
	No radiation	61 (59)
Chemotherapy	Chemotherapy	1 (1)
	Concurrent chemoradiation	3 (3)
	No chemotherapy	99 (96)
Disease status	No evidence of disease	64 (62)
	Recurrent disease	36 (35)
	Lost to follow-up	3 (3)
Outcome	Alive	61 (59)
	Tumor-related death	33 (32)
	Non tumor-related death	8 (8)
	Lost to follow-up	1 (1)

CI, confidence interval; ms, months; ND, not determined; SD, standard deviation; yrs, years.

^*^, includes 9 cases of concurent pelvic exenteration.

^**^, surgery was not performed due to death before surgery (2) or inoperable morbidity (2).

^***^, in 4 cases concurrent pelvic/paraaortic lymphadenectomy was performed.

^****^, in 12 cases concurrent pelvic/iliac radiation therapy was performed.

**Table 2:** Summary of staining and HPV analysis stratified by tissue compartment

Variable		Value [mean ± SD (range)]
CD3^+^ T cells (n)	Epithelium	23.31 ± 36.36 (0 - 143)
	Stroma	240.07 ± 357.42 (0.7 - 1478)
CD20^+^ B cells (n)	Epithelium	0.32 ± 2.46 (0 - 24)
	Stroma	46.58 ± 92.37 (0 - 503)
CD68^+^ monocytes/macrophages (n)	Epithelium	4.67 ± 8.32 (0 - 38)
	Stroma	22.97 ± 45 (0 - 378)
**Variable**		**Value [n (%)]**
PD-L1 immunoreactivity	Positive	10 (10)
	Negative	93 (90)
Ki-67 immunoreactivity	High	29 (28)
	Low	56 (54)
	ND	18 (18)
p53 immunoreactivity	Positive	21 (20)
	Negative	69 (67)
	ND	13 (13)
p16^INK4a^ immunoreactivity	Positive	30 (29)
	Negative	64 (62)
	ND	9 (9)
HPV high risk	Present	31 (30)
	Absent	69 (67)
	ND	3 (3)

ND, not determined; SD, standard deviation.

### VSCC express PD-L1 at variable degrees of intensity

**Figure 1:**
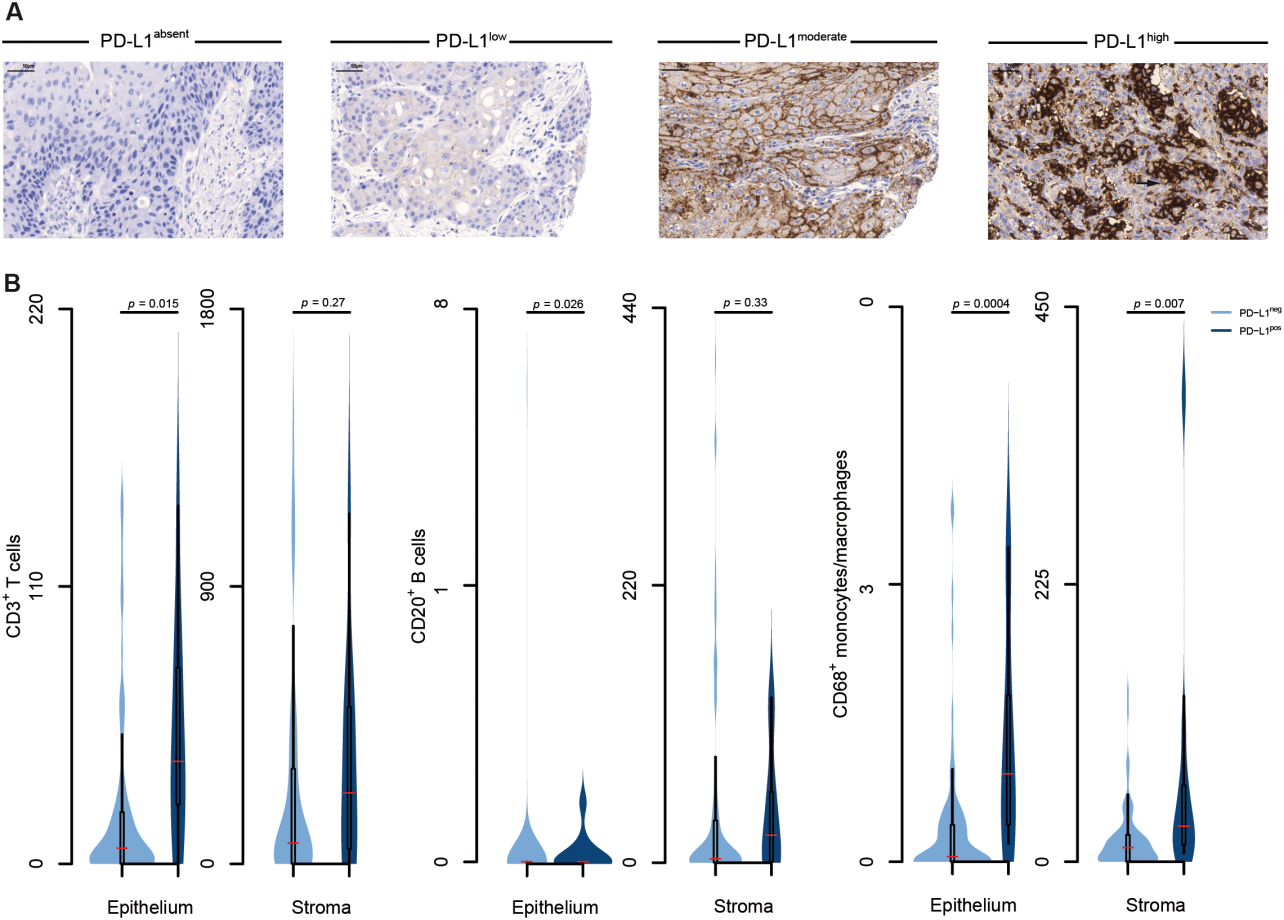
Identification of an immune microenvironment in VSCC **(A)** Representative images depict various expression levels of membranous PD-L1 expression in VSCC (brown cell membrane, arrow) visualized by immunohistochemistry; hematoxylin (blue) was used for nuclear staining (bright field image, 300× magnification). **(B)** Expression of PD-L1 was determined by immunohistochemistry in 103 patients with VSCC; immune cell populations were enumerated in the tumor (epithelial) and the peri-tumoral micromilieu (stroma) using immunohistochemistry; samples were divided into absent and present PD-L1 expression groups; frequencies of immune cells were calculated and plotted as number of cells per stromal and epithelial fraction, respectively, of the TMA; violin plots show the distribution of data points; the median, 25^th^ and 75^th^ percentiles and whiskers are depicted.

In the next step, we aimed to determine whether additional immune escape mechanisms of the tumor are present. Membranous expression of PD-L1 was identified in 23.3% (24/103) VSCC samples. The intensity of continuously stained cell membranes ranged from low (3 cases) to moderate (4 cases) to high (13 cases); four cases lacked confluence of staining (Figure 1A). The amount of stained cells varied between 5 and 60%; no positive case exhibited less than 5% of PD-L1 stained cells. To more closely reflect the biological reality we applied a stringent threshold. Using this cut-off, 9.7% (10/103) VSCCs were classified as PD-L1 positive (Table 2).

### Immune activation is associated with PD-L1 expression in VSCC

To examine the relationship between tumor PD-L1 and the immune microenvironment, VSCCs were divided according to their level of immune checkpoint expression. Then, we correlated PD-L1 with the presence of immune cells. We found its expression to be significantly related with CD3^+^, CD20^+^ and CD68^+^ intra-tumor immunocytes (Figure 1B). Numbers of CD3^+^ T cells, CD20^+^ B cells and CD68^+^ monocytes/macrophages were always higher in PD-L1-positive VSCCs. Additionally, the amount of stromal CD68^+^ monocytes/macrophages correlated proportionally with the intensity of tumor PD-L1 labeling. With regard to immunosuppressive cell populations, however, no correlation was observed between PD-L1 expression of the tumor and the amount of infiltrating T_reg_ cells and TAMs, respectively (Supplementary Figure 1). This was true for immunocytes found in tumor islets and stroma.

### PD-L1 expression is associated with HPV-negativity

**Figure 2:**
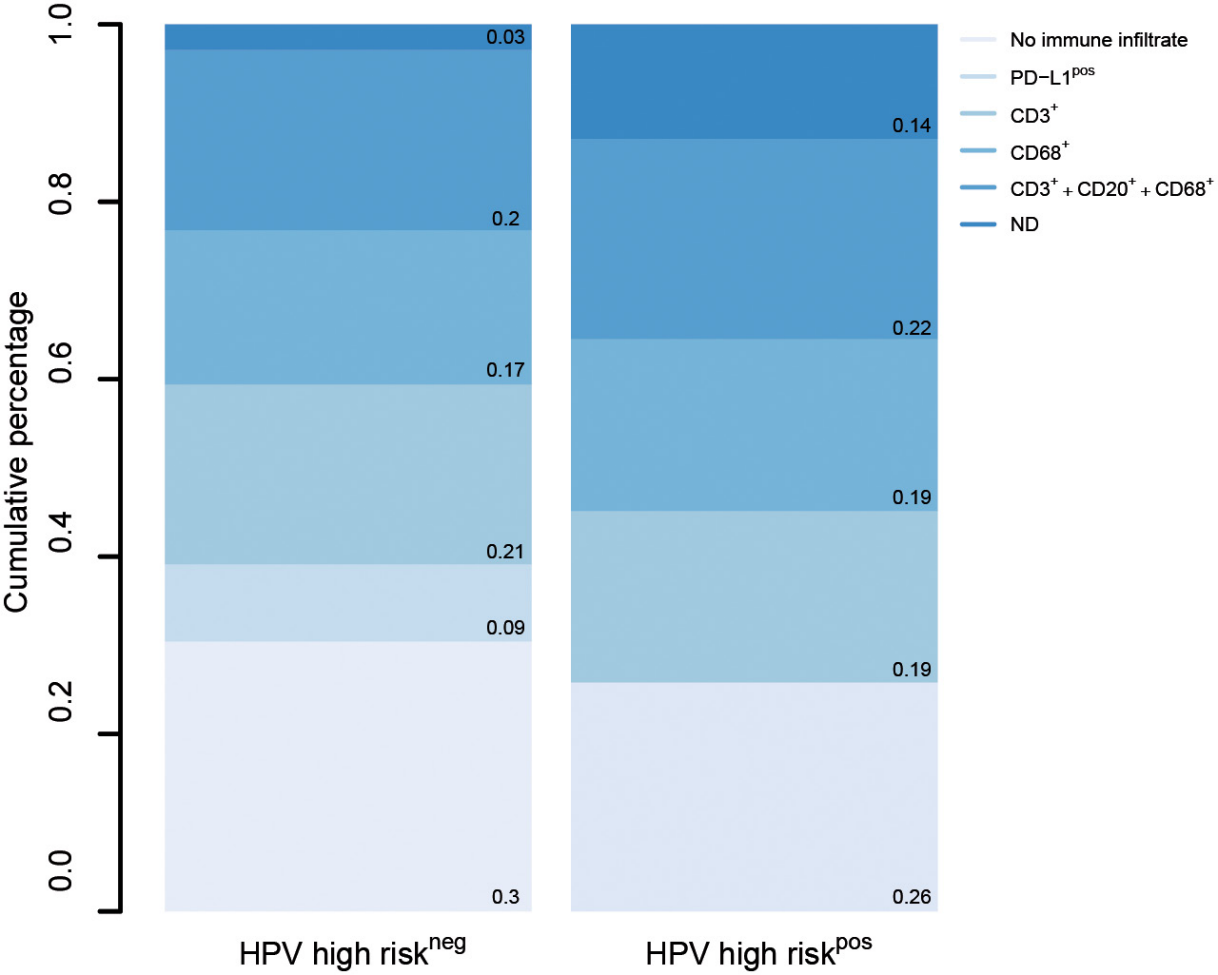
PD-L1 expression differs between HPV-positive and -negative cases Tumors were grouped according to HPV-status; stacked bar graphs illustrate relative proportions of immune parameters as determined by immunohistochemistry (immunocytes in the presence of PD-L1, infiltration of CD3^+^ or CD68^+^ cells only, concurrent immune infiltration of multiple immune cell types).

In order to better understand PD-L1-related tumor biology, clinicopathological parameters were correlated with the presence of PD-L1 expression. However, no significant associations were observed between PD-L1 presence and typical clinicopathological factors (Table 3). Based on the two pathogenic pathways known for VSCC, we then asked whether PD-L1 expression enriches in one entity. We determined (i) p53 immunoreactivity and the proliferation marker Ki-67 to identify VSCCs arising in the setting of *lichen sclerosus et atrophicus* [23]; (ii) high risk HPV and p16^INK4a^ (a marker of HPV-transforming activity) to identify VSCCs driven by viral infection [24]. We found PD-L1 expression to occur more often in high risk HPV-negative VSCCs (Table 4). This finding was confirmed by the observation that PD-L1 positivity also correlated with the absence of p16^INK4a^. With the exception of PD-L1, no difference was found for immune cells between viral and non-viral etiology (Figure 2).

**Table 3:** PD-L1 expression does not correlate with clincopathological parameters

Variable (number of patients evaluated)		PD-L1 expression		*p*-value
		Positive	Negative	
Tumor stage (99)	High	2	14	0.66
	Low	8	75	
	*NA*	*0*	*4*	
Depth of stromal invasion (87)	> 1mm	4	32	0.16
	≤ 1mm	1	50	
	*NA*	*5*	*11*	
Lymph node involvement (71)	Present	4	27	0.69
	Absent	3	37	
	*NA*	*3*	*29*	
Metastasis (99)	Present	1	4	0.42
	Absent	9	85	
	*NA*	*0*	*4*	
Tumor grade (102)	High	9	81	1.00
	Low	1	11	
	*NA*	*0*	*1*	
Lymphovascular space invasion (88)	Present	0	8	0.59
	Absent	10	70	
	*NA*	*0*	*15*	
Haemangioinvasion (88)	Present	0	6	1.00
	Absent	10	72	
	*NA*	*0*	*15*	

NA, not available.

**Table 4:** PD-L1 expression correlates with the absence of HPV

Variable (number of patients evaluated)		PD-L1 expression		*p*-value
		Positive	Negative	
Ki-67 immunoreactivity (85)	High	3	26	1.00
	Low	6	50	
	*NA*	*1*	*17*	
p53 immunoreactivity (90)	Positive	4	17	0.23
	Negative	6	63	
	*NA*	*0*	*13*	
p16^INK4a^ immunoreactivity (94)	Positive	0	30	0.028
	Negative	10	54	
	*NA*	*0*	*9*	
HPV high risk (100)	Present	0	31	0.029
	Absent	10	59	
	*NA*	*0*	*3*	

NA, not available.

### PD-L1 expression predicts impaired survival in VSCC

**Figure 3:**
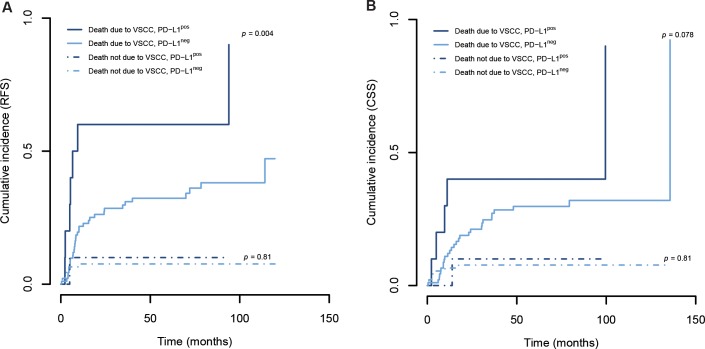
PD-L1 expression is a prognostic factor in VSCC The membranous expression of PD-L1 in cancer cells was determined by immunohistochemistry; cumulative incidence analysis was performed using competing risk regression (death from VSCC was the main event and death from other causes the competing risk approach); results of Gray's test are provided.

Given our observation that PD-L1 correlates with non-HPV pathogenesis, we analyzed whether the presence of PD-L1 would be of use to prognosticate poor outcome in VSCC. Since our cohort had an average age of 64 years, in which deaths from non-cancer illnesses are expected to occur in a higher proportion, the effect of mortality from causes other than VSCC was modeled as competing risk. PD-L1 expression was able to discriminate between recurrence-free survival (RFS) rates relating a positive staining to a significantly more unfavorable outcome (Figure 3A). Additionally, PD-L1 expression tended to be associated with a shorter cancer-specific survival (CSS, Figure 3B). We used Cox regression analysis and found the presence of PD-L1 also to be an independent prognostic factor for RFS (Table 5). Lymph node involvement is known to be the most important predictor of disease outcome and was used as the gold standard in our analysis [25–27]. Of note, PD-L1 expression enhanced the risk of relapse by 3.029 times, a hazard ratio (HR) comparable to that of lymphatic metastasis.

**Table 5:** Risk factors affecting recurrence-free and overall survival

	Recurrence-free survival					
Variable	Univariate analysis			Multivariate analysis^*^		
	HR	95% CI	*p*-value	HR	95% CI	*p*-value
PD-L1 immunoreactivity (positive vs. negative)	3.125	1.448 - 6.745	0.0037	3.029	1.228 - 7.471	0.0018
Lymph node involvement (present vs. absent)	3.076	1.546 - 6.119	0.0014	3.012	1.508 - 6.018	0.016
	**Overall survival**					
**Variable**	**Univariate analysis**			Multivariate analysis^*^		
	**HR**	**95% CI**	***p*-value**	**HR**	**95% CI**	***p*-value**
PD-L1 immunoreactivity (positive vs. negative)	2.222	0.928 - 5.32	0.07			
Lymph node involvement (present vs. absent)	5.038	2.28 - 11.13	0.00006			

CI, confidence interval; HR, Hazard ratio.

^*^Only variables significantly associated in the univariate analysis were included in the multivariate analysis.

## DISCUSSION

While immune checkpoint blockade may offer a promising novel therapeutic approach with regard to locally advanced, metastatic or recurrent disease, little is published about the immune landscape in VSCC and no data are available on the clinical relevance of PD-L1. We found PD-L1 to be expressed in VSCC within an immune-rich environment. The correlation of PD-L1 with HPV-negativity and poor outcome points towards immune escape mechanisms in a distinct subset of VSCC.

We observed CD3^+^ T cells, CD20^+^ B cells and CD68^+^ monocytes/macrophages as well as Foxp3^+^ T_reg_ cells and CD163^+^ TAMs to be present in peri- and intra-tumor areas. Data on the role that the immune system plays in VSCC are still scarce and conflicting. In accordance with our results, earlier studies have discovered evidence of CD4^+^ and CD8^+^ T cells in cancer nests and the peri-tumoral stroma [17–19]. However, their association with the clinical behavior of VSCC has remained largely unclear. A higher number of cytotoxic CD8^+^ T cells did not correlate with better outcome suggesting counteracting inhibitory mechanisms. In accordance with this hypothesis, higher levels of indoleamine 2,3-dioxygenase (IDO) expression were shown to be associated with poor outcome [28]. These findings, however, could not be replicated in a second study [18].

Our findings point towards a vital role of PD-L1 in the immune escape of VSCC. The observed correlation between tumor-associated immune cells and PD-L1 expression suggests that the VSCC-directed immune response provokes tolerance. The more stringent association with immunocytes in cancer cell islets is in accordance with previous data showing that intra-tumor leukocytes reflect immune reactions against cancer with higher accuracy [22]. Of note, a high density of CD8^+^ T cells at the tumor site itself was shown to be crucial for treatment response to PD-L1 blockade [29, 30]. The extensive occurrence of intra-tumor T cells in VSCC might thus recommend immune checkpoint blockade as a powerful therapeutic tool. In contrast to previous data, we failed to identify a significant relationship between immunosuppressive cell populations and PD-L1 expression in VSCC. PD-L1 has been shown to maintain the survival and to increase the suppressive activity of inducible T_reg_ cells [31]. Similarly, PD-L1 expression was observed to positively correlate with the amount of CD163^+^ TAMs in melanoma [32]. Potential reasons for divergent findings include inadequate VSCC sample size given the low expression of PD-L1, tumor site heterogeneity not represented by TMAs and the use of distinct antibodies.

Overall, we found PD-L1 to be present in VSCC, albeit at a low frequency. A previous study evaluated PD-L1 in a small number of 23 VSCCs [33]. However, the positivity rate was slightly higher (47.83%) when applying our threshold to their raw data. One reason for this conflicting result might be the use of a different antibody (9A11), which recognizes an epitope in the cytoplasmic domain of PD-L1, while our antibody targets an extracellular region [34, 35]. Authors also showed copy number gain of PD-1 ligands-encoding genes to occur commonly in VSCC. Unfortunately, their study lacked the evaluation of PD-L1-dependent clinical consequences.

We observed an unfavorable outcome in women with PD-L1-expressing tumors, suggesting impaired T cell-mediated immune responses and a microenvironment more permissive to tumor recurrence. Further support for this hypothesis comes from the observation that PD-L1 expression did not correlate with any commonly recognized feature of greater malignant potential. Our data suggest that PD-L1-based immune evasion mechanisms occur primarily in HPV-negative VSCCs. In penile cancer, PD-L1 expression was also found to be mainly expressed in HPV-negative tumors [36]. In Epstein-Barr-positive lymphoproliferative disorders and cervical cancer, however, viral infection represents a mechanism to provoke PD-L1 expression [37, 38]. In head and neck tumors conflicting results were published with regard to the HPV-dependence of PD-L1 [39, 40]. Thus, additional functional studies are needed to clarify the role HPV plays in PD-L1 induction.

Immune checkpoint blockade has been a game-changer in the field of tumor treatment and the identification of a biomarker that predicts the response to PD-L1 inhibition is essential. While PD-L1-negative tumors might also benefit from checkpoint inhibitors and some PD-L1-positive tumors do not respond to treatment, an increased probability of therapeutic response has been observed in tumors defined by the presence of PD-L1 [41, 42]. Accordingly, our observation of PD-L1 expression in the tumor bed of VSCC could be valuable in guiding clinical decision making. Additionally, our data suggest that the HPV status could potentially stratify patients for trials with anti-PD-L1 treatment.

To the best of our knowledge, this is the first study analyzing the clinical impact of PD-L1 expression in VSCC. The strength of our analysis lies in the compilation of immunological markers and diverse clinical as well as pathological data. A potential limitation of our study is the utilization of TMA. Although reliable for the determination of antigen expression, a heterogeneous protein distribution could potentially influence results [43]. In cervical squamous cell cancer, a tumor comparable to VSCC, both diffuse and focal expression of PD-L1 was observed [44]. Interestingly, women with homogeneously PD-L1-stained cervical malignancies showed poorer survival than those with a heterogeneous labeling. In the light of these data, we cannot rule out a TMA-dependent selection bias that favored diffuse PD-L1 expression. Our multivariate analysis identified PD-L1 expression as an independent prognostic factor of RFS but not of OS. Despite having a long-term follow-up, it may have been too short to identify independent prognostic factors of OS. Survival rates could have been influenced by (i) the study population with a high number of early stages; and (ii) therapy with curative intent in recurrent cases.

In summary, our study provides evidence that inhibitory pathways are operational in VSCC, arresting the tumor-specific immune response and influencing outcome. Our findings will hopefully boost the development of immunotherapy in VSCC. While further efforts must be made to validate our findings of PD-L1 as a prognostic factor and determine its value as a predictive biomarker, checkpoint blockade could be the leading path to increased treatment success in VSCC.

## MATERIALS AND METHODS

### Patients and specimens

The retrospective study population included 103 patients with primary VSCC diagnosed at the University of Bonn between 2002 and 2013. Women who underwent neoadjuvant treatment were excluded. Patients were treated according to the S2k guideline of the German Cancer Society and the German Society for Gynecology and Obstetrics. Our cohort had a high number of women not treated with lymph node dissection. Due to cardiovascular comorbidities, 20 patients with early-stage tumors (pTIb/II) did not undergo lymphonodectomy; clinically and sonographically lymph nodes appeared to be unaffected. Additionally, the presence of lymphovascular space invasion was recorded, known to be a risk factor for lymph node metastasis [45]. In our cohort, however, no significant association between lymph node involvement and lymphovascular space invasion was noted, likely due to the fact that the detection is challenging in the absence of lymphatic endothelium-specific markers [46]. Baseline characteristics were obtained from a clinical database. Follow-up data were updated until August 2016. Histopathological diagnosis was made based on World Health Organization (WHO) criteria. The 2010 revision of the International Federation of Gynecology and Obstetrics (FIGO) system was used to assign the tumor grade; the 7^th^ TNM classification of the Union for International Cancer Control (UICC) was used to determine the tumor stage. For tumors diagnosed before 2009, stages were re-classified according to the updated version. The study was approved by the Institutional Review Board of the Medical Faculty of the University of Bonn, Germany (228/15).

### Tissue microarray (TMA) construction

The TMA was created from archival formalin-fixed paraffin-embedded tissue (FFPE) specimens. Sections stained with hematoxylin and eosin (HE) were used to identify representative tumor areas. For each case, one 1 mm core biopsy (0.785mm^2^) was taken from the selected cancer nests and arranged in TMA blocks.

### Immunohistochemistry

Immunostaining of PD-L1, CD3, CD20 and CD68 was performed on TMAs using an automated staining system (BenchMark ULTRA; Ventana Medical Systems, Tucson, Arizona, USA) and the Ventana amplifier detection kit for visualization. Additionally, in a subset of our cohort (42 samples) staining of CD163 and Foxp3 was performed on serial 4μm sections using an automated staining system (DAKO TechMate 500; DAKO, Glostrup, Denmark) and the streptavidin-biotin-peroxidase/DAB technique (DAKO) for visualization. To determine PD-L1 expression we employed an antibody that is FDA-approved for selecting non-small cell lung cancer patients for treatment with the anti-PD-1 antibody pembrolizumab [35]. Primary antibodies included the following: mouse anti-human PD-L1 IgG1 monoclonal antibody (clone 22C3, dilution1:25; Dako, Glostrup, Denmark), mouse anti-human Ki-67 IgG1 monoclonal antibody (clone MIB-1, dilution 1:500; Dako), mouse anti-human p53 IgG2b monoclonal antibody (clone DQ-7, dilution 1:500; Dako), CINtec^©^ Histology kit for the evaluation of p16^INK4a^ (Roche, Basel, Switzerland), mouse anti-human CD3 IgG2a monoclonal antibody (clone PS1, dilution 1:50; Novocastra, Newcastle Upon Tyne, United Kingdom), mouse anti-human CD20 IgG2a monoclonal antibody (clone L26, dilution 1:2000; Dako), mouse anti-human CD68 IgG3 monoclonal antibody (clone PG-M1, dilution 1:250, Dako), mouse anti-human CD163 IgG1 monoclonal antibody (clone 10D6, dilution 1: 1250, Leica Biosystems, Wetzlar, Germany), mouse anti-human Foxp3 IgG1 monoclonal antibody (clone 236A/E7, dilution 1: 50, Abcam, Cambridge, UK).

### Evaluation of immunoreactions

Immunostained cells were analyzed with a Leica DM LB2 microscope (Leica Microsystems, Wetzlar, Germany) with the Pannoramic Viewer (3DHISTECH Ltd., Budapest, Hungary) or the Axio Observer D1 microscope (Zeiss, Jena, Germany) with the AxioVision 4.7 software (Zeiss). As a negative control for PD-L1 staining, we used sections of kidney, as a positive control sections of tonsil (Supplementary Figure 2). The membranous expression of PD-L1 on tumor cells was scored positive only if the immunoreactivity showed a honeycomb pattern (i.e. continuously stained cell membranes); cytoplasmic staining of PD-L1 was disregarded. Our analysis is based on an integrated proportion score that aims to harmonize different PD-L1 immunoassays [47]. In detail, a four-tier scoring system was applied to categorize the staining intensity (0, no staining; 1, low staining; 2, moderate staining; 3, high staining). Additionally, the percentage of PD-L1-stained tumor cells was estimated (0, <1%; 1, ≥1%; 2 ≥5%; 3, ≥10%; 4, ≥25%; 5, ≥50%). Malignancies with a staining intensity ≥ 2 in ≥ 5% of tumor cells were regarded as PD-L1 positive [48]. For Ki-67, a nuclear staining of cancer cells was interpreted as positive and the percentage of labeled cells in the vulvar epithelium was recorded; for p53, a nuclear staining in >25% of tumor cells was graded positive [49, 50]; for p16^INK4a^, a continuous strong nuclear plus cytoplasmic labeling of the basal cells with extension upward involving ≥30% of the epithelial thickness was regarded as positive [49, 51, 52]. Immune cell populations were determined separately in the epithelium and the stroma. T cells were identified by membranous and cytoplasmic expression of CD3, B cells by membranous and cytoplasmic expression of CD20, monocytes/macrophages by cytoplasmic expression of CD68, TAMs by membranous and cytoplasmic expression of CD163, T_reg_ cells by nuclear staining of Foxp3. CD3^+^ T cells, CD20^+^ B cells and CD68^+^ monocytes/macrophages were enumerated using the whole core area of the TMA (values are given as the number of immune cells per stromal and epithelial fraction of the TMA, respectively). T_reg_ cells and TAMs were determined in three high-power fields (HPFs) with maximum infiltration and set in relation to the relative amount of tumor and stroma of these HPFs (values are given as the mean percentage of immune cells per stromal and epithelial fraction of individual HPFs). T_reg_ cells were enumerated; TAMs were recorded digitally using a semi-automated computerized method as described before [21].

### DNA extraction

After deparaffinization, tumor tissue was macrodissected from unstained slides and tissue was lysed with proteinase K overnight. Subsequently, extraction of DNA from FFPE-embedded tissue DNA was carried out with the BioRobot M48 Robotic Workstation and the corresponding MagAttract DNA Mini M48 Kit (Qiagen, Hilden, Germany) following the manufacturer's protocol. Quality of genomic DNA was assessed by agarose gel electrophoresis. DNA concentration of each sample was determined using Nanodrop 2000 (PeqLab, Erlangen, Germany).

### HPV analysis

The HPV Type 3.5 LCD-Array Kit was used for the determination of HPV subtypes by hybridization to HPV-specific DNA probes (Chipron, Berlin, Germany). Amplification of HPV-specific DNA segments (L1 region) was achieved using the primer sets HPV ‘125′ and HPV MY09/MY11. Ten microliters of the amplification products were hybridized to HPV type-specific capture probes fixed to an LCD array chip. All steps were performed according to manufacturer's recommendations.

### Statistical analysis

Statistical analysis was carried out using ‘R’ version 3.2.3 (The R Foundation for Statistical Computing, Vienna, Austria). Comparisons between PD-L1 expression and continuous data were performed using the Kruskal-Wallis test; comparisons between PD-L1 expression (positive, negative) and categorical variables using the Fisher's exact test. Ki-67 staining in ≥ 30% of the epithelium was used as a cut-off point to assign tumors into high and low reactivity groups [23]. Poor-differentiation - grades 2,3 [53] - and advanced tumor stages - stages pT2, 3 [54] - have been shown to result in a poorer prognosis and, thus, thresholds were set between grades 2,3 and 1 and between stages pT2, 3 and pT1, respectively. Cumulative incidence analysis (RFS, CSS) was modeled using the competing risk approach and curves were compared with Gray's test [55]. Multivariate survival analysis (RFS, OS) was done using the Cox's proportional hazard regression model. The confidence interval for the median follow-up time was calculated using quantiles of the binomial distribution.

## SUPPLEMENTARY MATERIALS FIGURES AND TABLE



## Abbreviations

VSCC: squamous cell carcinoma
HPV: human papillomavirus
PD-1: programmed death 1 receptor
PD-L1: programmed death ligand 1
IDO: indoleamine 2,3-dioxygenase
HR: hazard ratio
RFS: recurrence-free survival
CSS: cancer-specific survival
OS: overall survival
FFPE: formalin-fixed paraffin-embedded tissue
HE: hematoxylin and eosin
TMA: tissue microarray
TAM: tumor-associated macrophage
HPF: high-power field
T_reg_ cell: regulatory T cell
WHO: World Health Organization criteria
FIGO: International Federation of Gynecology and Obstetrics
UICC: Union for International Cancer Control

## References

[1] Siegel RL, Miller KD, Jemal A (2016). Cancer statistics, 2016. CA Cancer J Clin.

[2] Lai J, Elleray R, Nordin A, Hirschowitz L, Rous B, Gildea C, Poole J (2014). Vulval cancer incidence, mortality and survival in England: age-related trends. BJOG.

[3] Baandrup L, Varbo A, Munk C, Johansen C, Frisch M, Kjaer SK (2011). *In situ* and invasive squamous cell carcinoma of the vulva in Denmark 1978-2007-a nationwide population-based study. Gynecol Oncol.

[4] Schuurman MS, van den Einden LC, Massuger LF, Kiemeney LA, van der Aa MA, de Hullu JA (2013). Trends in incidence and survival of Dutch women with vulvar squamous cell carcinoma. Eur J Cancer.

[5] van der Avoort IA, Shirango H, Hoevenaars BM, Grefte JM, de Hullu JA, de Wilde PC, Bulten J, Melchers WJ, Massuger LF (2006). Vulvar squamous cell carcinoma is a multifactorial disease following two separate and independent pathways. Int J Gynecol Pathol.

[6] Insinga RP, Liaw KL, Johnson LG, Madeleine MM (2008). A systematic review of the prevalence and attribution of human papillomavirus types among cervical, vaginal, and vulvar precancers and cancers in the United States. Cancer Epidemiol Biomarkers Prev.

[7] Akhtar-Danesh N, Elit L, Lytwyn A (2014). Trends in incidence and survival of women with invasive vulvar cancer in the United States and Canada: a population-based study. Gynecol Oncol.

[8] Rauh-Hain JA, Clemmer J, Clark RM, Bradford LS, Growdon WB, Goodman A, Boruta DM, Dizon DS, Schorge JO, del Carmen MG (2014). Management and outcomes for elderly women with vulvar cancer over time. BJOG.

[9] Howlader N, Noone AM, Krapcho M, Miller D, Bishop K, Altekruse SF, Kosary CL, Yu MR, Tatalovich Z, Mariotto A, Lewis DR, Chen HS, Feuer EJ (2016). SEER Cancer Statistics Review, 1975-2013.

[10] Clancy AA, Spaans JN, Weberpals JI (2016). The forgotten woman's cancer: vulvar squamous cell carcinoma (VSCC) and a targeted approach to therapy. Ann Oncol.

[11] Keir ME, Butte MJ, Freeman GJ, Sharpe AH (2008). PD-1 and its ligands in tolerance and immunity. Annu Rev Immunol.

[12] Hino R, Kabashima K, Kato Y, Yagi H, Nakamura M, Honjo T, Okazaki T, Tokura Y (2010). Tumor cell expression of programmed cell death-1 ligand 1 is a prognostic factor for malignant melanoma. Cancer.

[13] Hamanishi J, Mandai M, Iwasaki M, Okazaki T, Tanaka Y, Yamaguchi K, Higuchi T, Yagi H, Takakura K, Minato N, Honjo T, Fujii S (2007). Programmed cell death 1 ligand 1 and tumor-infiltrating CD8+ T lymphocytes are prognostic factors of human ovarian cancer. Proc Natl Acad Sci U S A.

[14] Mu CY, Huang JA, Chen Y, Chen C, Zhang XG (2011). High expression of PD-L1 in lung cancer may contribute to poor prognosis and tumor cells immune escape through suppressing tumor infiltrating dendritic cells maturation. Med Oncol.

[15] Thompson RH, Kuntz SM, Leibovich BC, Dong H, Lohse CM, Webster WS, Sengupta S, Frank I, Parker AS, Zincke H, Blute ML, Sebo TJ, Cheville JC (2006). Tumor B7-H1 is associated with poor prognosis in renal cell carcinoma patients with long-term follow-up. Cancer Res.

[16] Sunshine J, Taube JM (2015). PD-1/PD-L1 inhibitors. Curr Opin Pharmacol.

[17] Sznurkowski JJ, Zawrocki A, Emerich J, Biernat W (2011). Prognostic significance of CD4+ and CD8+ T cell infiltration within cancer cell nests in vulvar squamous cell carcinoma. Int J Gynecol Cancer.

[18] de Jong RA, Toppen NL, Ten Hoor KA, Boezen HM, Kema IP, Hollema H, Nijman HW (2012). Status of cellular immunity lacks prognostic significance in vulvar squamous carcinoma. Gynecol Oncol.

[19] Sznurkowski JJ, Zawrocki A, Biernat W (2014). Subtypes of cytotoxic lymphocytes and natural killer cells infiltrating cancer nests correlate with prognosis in patients with vulvar squamous cell carcinoma. Cancer Immunol Immunother.

[20] Patel SP, Kurzrock R (2015). PD-L1 expression as a predictive biomarker in cancer immunotherapy. Mol Cancer Ther.

[21] Kübler K, Ayub TH, Weber SK, Zivanovic O, Abramian A, Keyver-Paik MD, Mallmann MR, Kaiser C, Serce NB, Kuhn W, Rudlowski C (2014). Prognostic significance of tumor-associated macrophages in endometrial adenocarcinoma. Gynecol Oncol.

[22] Pölcher M, Braun M, Friedrichs N, Rudlowski C, Bercht E, Fimmers R, Sauerwald A, Keyver-Paik MD, Kubler K, Buttner R, Kuhn WC, Hernando JJ (2010). Foxp3(+) cell infiltration and granzyme B(+)/Foxp3(+) cell ratio are associated with outcome in neoadjuvant chemotherapy-treated ovarian carcinoma. Cancer Immunol Immunother.

[23] Hoevenaars BM, van der Avoort IA, de Wilde PC, Massuger LF, Melchers WJ, de Hullu JA, Bulten J (2008). A panel of p16(INK4A), MIB1 and p53 proteins can distinguish between the 2 pathways leading to vulvar squamous cell carcinoma. Int J Cancer.

[24] Halec G, Alemany L, Quiros B, Clavero O, Hofler D, Alejo M, Quint W, Pawlita M, Bosch FX, de Sanjose S (2017). Biological relevance of human papillomaviruses in vulvar cancer. Mod Pathol.

[25] Maggino T, Landoni F, Sartori E, Zola P, Gadducci A, Alessi C, Solda M, Coscio S, Spinetti G, Maneo A, Ferrero A, Konishi De Toffoli G (2000). Patterns of recurrence in patients with squamous cell carcinoma of the vulva. A multicenter CTF Study. Cancer.

[26] Burger MP, Hollema H, Emanuels AG, Krans M, Pras E, Bouma J (1995). The importance of the groin node status for the survival of T1 and T2 vulval carcinoma patients. Gynecol Oncol.

[27] Braun M, Wardelmann E, Debald M, Walgenbach-Bruenagel G, Holler T, Wolfgarten M, Sauerwald A, Rudlowski C, Buttner R, Kuhn W, Polcher M (2009). Detection of lymphovascular invasion in vulvar cancer by D2-40 (podoplanin) as a predictor for inguinal lymph node metastases. Onkologie.

[28] Sznurkowski JJ, Zawrocki A, Emerich J, Sznurkowska K, Biernat W (2011). Expression of indoleamine 2,3-dioxygenase predicts shorter survival in patients with vulvar squamous cell carcinoma (vSCC) not influencing on the recruitment of FOXP3-expressing regulatory T cells in cancer nests. Gynecol Oncol.

[29] Tumeh PC, Harview CL, Yearley JH, Shintaku IP, Taylor EJ, Robert L, Chmielowski B, Spasic M, Henry G, Ciobanu V, West AN, Carmona M, Kivork C (2014). PD-1 blockade induces responses by inhibiting adaptive immune resistance. Nature.

[30] Tang H, Wang Y, Chlewicki LK, Zhang Y, Guo J, Liang W, Wang J, Wang X, Fu YX (2016). Facilitating T cell infiltration in tumor microenvironment overcomes resistance to PD-L1 blockade. Cancer Cell.

[31] Francisco LM, Salinas VH, Brown KE, Vanguri VK, Freeman GJ, Kuchroo VK, Sharpe AH (2009). PD-L1 regulates the development, maintenance, and function of induced regulatory T cells. J Exp Med.

[32] Obeid JM, Erdag G, Smolkin ME, Deacon DH, Patterson JW, Chen L, Bullock TN, Slingluff CL (2016). PD-L1, PD-L2 and PD-1 expression in metastatic melanoma: correlation with tumor-infiltrating immune cells and clinical outcome. Oncoimmunology.

[33] Howitt BE, Sun HH, Roemer MG, Kelley A, Chapuy B, Aviki E, Pak C, Connelly C, Gjini E, Shi Y, Lee L, Viswanathan A, Horowitz N (2016). Genetic Basis for PD-L1 Expression in Squamous Cell Carcinomas of the Cervix and Vulva. JAMA Oncol.

[34] Mahoney KM, Sun H, Liao X, Hua P, Callea M, Greenfield EA, Hodi FS, Sharpe AH, Signoretti S, Rodig SJ, Freeman GJ (2015). PD-L1 antibodies to its cytoplasmic domain most clearly delineate cell membranes in immunohistochemical staining of tumor cells. Cancer Immunol Res.

[35] Sholl LM, Aisner DL, Allen TC, Beasley MB, Borczuk AC, Cagle PT, Capelozzi V, Dacic S, Hariri L, Kerr KM, Lantuejoul S, Mino-Kenudson M, Raparia K (2016). Programmed death ligand-1 immunohistochemistry--a new challenge for pathologists: a perspective from members of the pulmonary pathology society. Arch Pathol Lab Med.

[36] Ottenhof SR, Djajadiningrat RS, de Jong J, Thygesen HH, Horenblas S, Jordanova ES (2017). Expression of Programmed Death Ligand 1 (PD-L1) in penile cancer is of prognostic value and associated with HPV status. J Urol.

[37] Green MR, Rodig S, Juszczynski P, Ouyang J, Sinha P, O'Donnell E, Neuberg D, Shipp MA (2012). Constitutive AP-1 activity and EBV infection induce PD-L1 in Hodgkin lymphomas and posttransplant lymphoproliferative disorders: implications for targeted therapy. Clin Cancer Res.

[38] Mezache L, Paniccia B, Nyinawabera A, Nuovo GJ (2015). Enhanced expression of PD L1 in cervical intraepithelial neoplasia and cervical cancers. Mod Pathol.

[39] Kim HR, Ha SJ, Hong MH, Heo SJ, Koh YW, Choi EC, Kim EK, Pyo KH, Jung I, Seo D, Choi J, Cho BC, Yoon SO (2016). PD-L1 expression on immune cells, but not on tumor cells, is a favorable prognostic factor for head and neck cancer patients. Sci Rep.

[40] Kim HS, Lee JY, Lim SH, Park K, Sun JM, Ko YH, Baek CH, Son YI, Jeong HS, Ahn YC, Lee MY, Hong M, Ahn MJ (2016). Association between PD-L1 and HPV status and the prognostic value of PD-L1 in oropharyngeal squamous cell carcinoma. Cancer Res Treat.

[41] Lipson EJ, Forde PM, Hammers HJ, Emens LA, Taube JM, Topalian SL (2015). Antagonists of PD-1 and PD-L1 in cancer treatment. Semin Oncol.

[42] Topalian SL, Hodi FS, Brahmer JR, Gettinger SN, Smith DC, McDermott DF, Powderly JD, Carvajal RD, Sosman JA, Atkins MB, Leming PD, Spigel DR, Antonia SJ (2012). Safety, activity, and immune correlates of anti-PD-1 antibody in cancer. N Engl J Med.

[43] Fons G, van der Velden J, Burger M, ten Kate F (2009). Validation of tissue microarray technology in vulvar cancer. Int J Gynecol Pathol.

[44] Heeren AM, Punt S, Bleeker MC, Gaarenstroom KN, van der Velden J, Kenter GG, de Gruijl TD, Jordanova ES (2016). Prognostic effect of different PD-L1 expression patterns in squamous cell carcinoma and adenocarcinoma of the cervix. Mod Pathol.

[45] Homesley HD, Bundy BN, Sedlis A, Yordan E, Berek JS, Jahshan A, Mortel R (1991). Assessment of current International Federation of Gynecology and Obstetrics staging of vulvar carcinoma relative to prognostic factors for survival (a Gynecologic Oncology Group study). Am J Obstet Gynecol.

[46] Weber SK, Sauerwald A, Polcher M, Braun M, Debald M, Serce NB, Kuhn W, Brunagel-Walgenbach G, Rudlowski C (2012). Detection of lymphovascular invasion by D2-40 (podoplanin) immunoexpression in endometrial cancer. Int J Gynecol Cancer.

[47] Scheel AH, Dietel M, Heukamp LC, Johrens K, Kirchner T, Reu S, Ruschoff J, Schildhaus HU, Schirmacher P, Tiemann M, Warth A, Weichert W, Fischer RN (2016). Harmonized PD-L1 immunohistochemistry for pulmonary squamous-cell and adenocarcinomas. Mod Pathol.

[48] Meng X, Huang Z, Teng F, Xing L, Yu J (2015). Predictive biomarkers in PD-1/PD-L1 checkpoint blockade immunotherapy. Cancer Treat Rev.

[49] Santos M, Landolfi S, Olivella A, Lloveras B, Klaustermeier J, Suarez H, Alos L, Puig-Tintore LM, Campo E, Ordi J (2006). p16 overexpression identifies HPV-positive vulvar squamous cell carcinomas. Am J Surg Pathol.

[50] Pinto AP, Miron A, Yassin Y, Monte N, Woo TY, Mehra KK, Medeiros F, Crum CP (2010). Differentiated vulvar intraepithelial neoplasia contains Tp53 mutations and is genetically linked to vulvar squamous cell carcinoma. Mod Pathol.

[51] Pirog EC (2015). Immunohistochemistry and in situ hybridization for the diagnosis and classification of squamous lesions of the anogenital region. Semin Diagn Pathol.

[52] Darragh TM, Colgan TJ, Cox JT, Heller DS, Henry MR, Luff RD, McCalmont T, Nayar R, Palefsky JM, Stoler MH, Wilkinson EJ, Zaino RJ, Wilbur DC (2012). The lower anogenital squamous terminology standardization project for HPV-associated lesions: background and consensus recommendations from the College of American Pathologists and the American Society for Colposcopy and Cervical Pathology. Arch Pathol Lab Med.

[53] Sznurkowski JJ, Zawrocki A, Biernat W (2016). The overexpression of p16 is not a surrogate marker for high-risk human papilloma virus genotypes and predicts clinical outcomes for vulvar cancer. BMC Cancer.

[54] Woelber L, Mahner S, Voelker K, Eulenburg CZ, Gieseking F, Choschzick M, Jaenicke F, Schwarz J (2009). Clinicopathological prognostic factors and patterns of recurrence in vulvar cancer. Anticancer Res.

[55] Fine JP, Gray RJ (2012). A proportional hazards model for the subdistribution of a competing risk. J Am Stat Assoc.

