# Supplementary material for: Tumoral PD-L1 expression defines a subgroup of poor-prognosis vulvar carcinomas with non-viral etiology
Source: Oncotarget. 2017 Oct 6;8(54):92890–903. doi: 10.18632/oncotarget.21641 (PMC5696230; doi:10.18632/oncotarget.21641)
Supplement: Supplementary file 1 [file oncotarget-08-92890-s001.pdf]

Tumoral PD-L1 expression defines a subgroup of poor-prognosis vulvar carcinomas with non-viral etiology

SUPPLEMENTARY MATERIALS

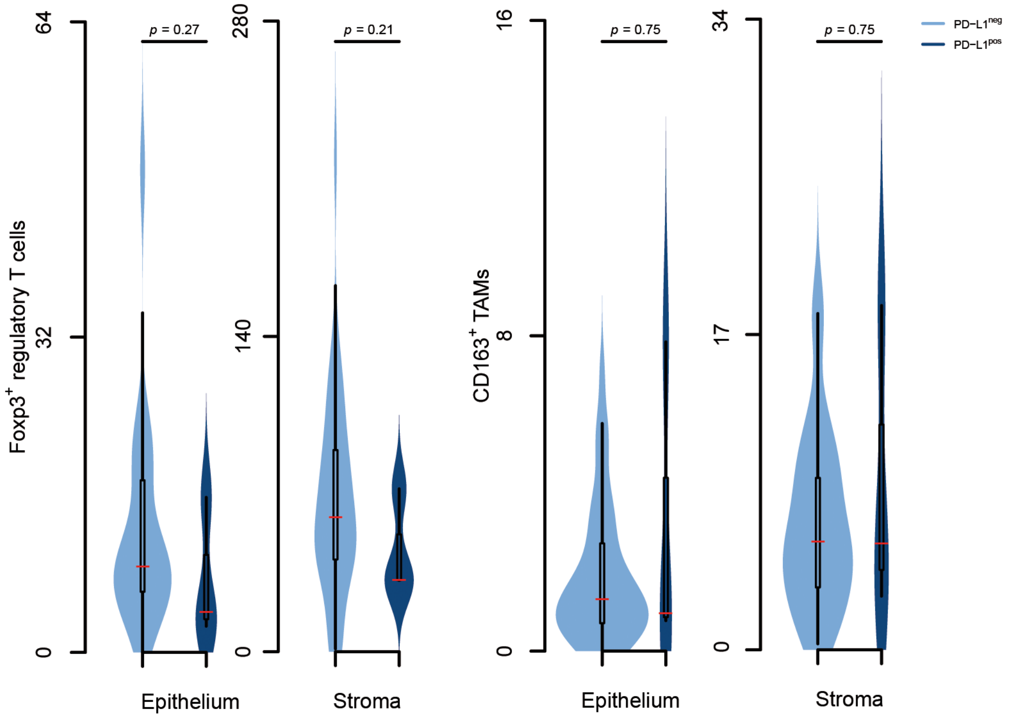

Supplementary Figure 1: Immunosuppressive cell populations are not associated with PD-L1 expression. Expression of PD-L1 was determined by immunohistochemistry; immunosuppressive cell populations were determined in the tumor (epithelial) and the peri-tumoral micromilieu (stroma) using immunohistochemistry; samples were divided into absent and present PD-L1 expression groups; values depict the mean percentage of numbers (Foxp3^{+} T_{reg} cells) and immunoreactivity (CD163^{+} TAMs), respectively, per tissue compartment (tumor or stroma); violin plots depict the distribution of data points; the median, 25th and 75th percentiles and whiskers are shown.

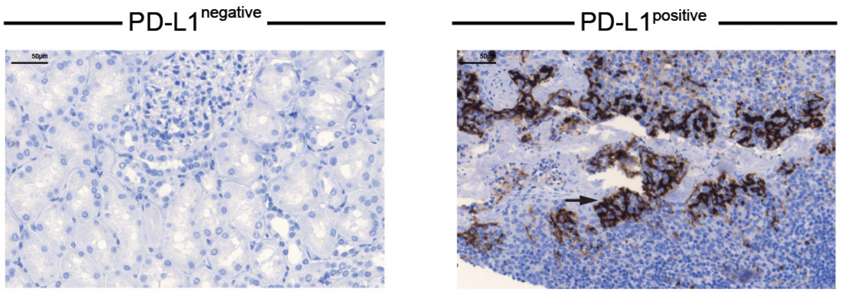

Supplementary Figure 2: PD-L1 staining controls. Representative images depict expression of membranous PD-L1 in kidney tissue (negative control) and tonsil tissue (positive control, brown cell membrane, arrow) visualized by immunohistochemistry; hematoxylin (blue) was used for nuclear staining (bright field image, 300× magnification).

Supplementary Table 1: Summary of staining of immunosuppressive cell populations

Variable		Value [mean ± SD (range)]
$Foxp3^+$ regulatory T cells (n)	Epithelium	11.99 ± 10.47 (0 - 50.83)
	Stroma	65.32 ± 42.42 (1.3 - 219.44)
$CD163^+$ TAMs (immunoreactivity)	Epithelium	1.98 ± 1.90 (0 - 7.85)
	Stroma	7.01 ± 4.94 (0.33 - 19.29)

SD, standard deviation; TAMs, tumor-associated macrophages.
